# Wavering between life and death: a qualitative study into the perceived causes and needs of persons with persistent suicidality

**DOI:** 10.3389/fpsyt.2025.1664180

**Published:** 2025-10-24

**Authors:** Mette Palm, Sisco van Veen, Lizanne Schweren, Aartjan Beekman, Els van Wijngaarden, Elke Elzinga

**Affiliations:** ^1^ Department of Psychiatry, Location Vrije Universiteit Amsterdam, Amsterdam University Medical Center, Amsterdam, Netherlands; ^2^ Mental Health Program, Amsterdam Public Health, Amsterdam, Netherlands; ^3^ Stichting 113 Zelfmoordpreventie, Amsterdam, Netherlands; ^4^ Department of Anesthesiology, Pain and Palliative Medicine, Radboud University Medical Center, Nijmegen, Netherlands

**Keywords:** persistent suicidality, chronic suicidality, perceived causes, needs, persistent suicidal ideation, lived experience

## Abstract

**Background:**

One of the key issues in the current knowledge base on persistent or chronic suicidality is the discrepancy between the extensive experiential knowledge embedded in clinical practice and its limited representation in the scientific literature. Both individuals living with persistent suicidality and the professionals who support them develop beliefs about its underlying causes and what is needed for effective management or treatment. Studying these subjective understandings may provide valuable insights into the contextual, relational and psychological factors related to persistent suicidality and help inform appropriate care strategies.

**Aims:**

This study aims to identify the perceived causes of persistent suicidality and the needs of those affected, from both the perspectives of individuals with lived experience of persistent suicidality and professionals providing support.

**Method:**

Semi-structured interviews were conducted with 12 individuals with lived experience of persistent suicidality and 10 occupational experts. Data were analyzed using reflexive thematic analysis.

**Results:**

Although individual experiences vary, persistent suicidality is perceived to be linked to childhood trauma and psychopathology. It may persist due to its perceived functionality or as a result of unsuccessful treatment and resulting feelings of demoralization. Key needs include continuity in care relationships, feeling genuinely heard, and openly discussing suicidal thoughts. This involves acceptance of these thoughts and exploring their meaning. Some individuals with persistent suicidality perceive aspects of care as ineffective or, in some cases, as contributing to further distress.

**Conclusions:**

Care for individuals with persistent suicidality should not focus solely on recovery but also on the acceptance and managing of suicidal thoughts. Effective care strategies may include teaching alternative coping and communication methods. The findings underscore the need for continuity of care and emphasize the importance of addressing non-disclosure and transference-countertransference dynamics in therapy.

## 1 Introduction

Suicide ranks among the leading causes of death worldwide, accounting for more than one in every 100 deaths (1.1%) in 2021 (1). Almost one in ten (9.2%) individuals experience suicidal ideation at some point in their lives (2). When individuals experience suicidal ideation, the initial response is typically to offer immediate assistance and to ensure safety, sometimes coercively (3). This can be very effective since a death wish often arises in response to psychological distress or significant social challenges, and treatments are available (4–6). However, there is a considerable group for whom the death wish does not decrease but persists.

In recent years, a growing body of scientific literature has turned its attention to the phenomenon of long-term forms of suicidality, also known as chronic or *persistent suicidality*. As there is no universally agreed-upon definition of suicidality, this study conceptualizes it as a broad spectrum, ranging from death wishes and suicidal plans to suicide attempts and death by suicide (7). Relatedly, well operationalized and empirically tested definitions or criteria for persistent suicidality do not yet exist and therefore cannot be presented. Nonetheless, the literature sometimes characterizes it as the presence of suicide ideation for a period of at least one year (8, 9).

Despite the early stage of research on persistent suicidality, research findings indicate that 28-43% of people who consider suicide at some point still do so years later (10–13). Prior studies suggest that individuals with persistent suicidality experience poorer psychological and mental health outcomes compared to those with no history of suicidality or transient suicidal ideation (8, 14, 15). A particularly elevated occurrence is observed in individuals with borderline personality disorder and affective disorders (12, 16, 17). In addition, persistent suicidality has been linked to various factors, among which early life adversity (18), high levels of hopelessness (12), sleeping problems (8, 12) and lower educational and income levels (13).

One of the key issues in the current knowledge base on persistent suicidality is the discrepancy between the considerable amount of knowledge held by care professionals and the limited representation of this knowledge in the scientific literature. There is limited understanding of how persistent suicidality is perceived by both care providers and individuals with lived experience. Most existing studies on persistent suicidality rely on quantitative methods, with insights predominantly shaped by external observers who typically lack the lived experience (19). Both individuals living with long-term conditions and those providing care and support have been found to develop subjective understandings of the condition, shaped by their experiences and beliefs (20–22). They formulate causal beliefs about why a condition developed and how it can be controlled or treated. These understandings shape how patients deal with their condition and how caregivers diagnose, treat, and assess risk, which is particularly of importance in cases of suicidality (20–23). Studying these subjective understandings of persistent suicidality may offer valuable new insights into the contextual and psychological factors related to it. To our knowledge, no prior study has qualitatively explored the experiences and perceptions of individuals living with persistent suicidality or those offering care and support for them.

This study aimed to identify perceived causes of persistent suicidality and the needs of those enduring it, from the perspective of both individuals living with persistent suicidality and those providing care and support. In doing so, it sought to complement the limited scientific literature with knowledge derived from clinical practice. Perceived causes and needs can be deeply interconnected, with specific causes directly influencing individual needs. Studying them together provides a broad understanding of the scope of challenges related to persistent suicidality. Findings may inform recommendations for appropriate responses in the prevention or treatment of persistent suicidality, as well as inform future research directions.

## 2 Methods

### 2.1 Study design

Semi-structured interviews were used to gather insights into the perceived causes of persistent suicidality and the needs of individuals with persistent suicidality, from the perspective of both experts by occupation (EbyO) and experts with lived experience (EbyE). This study adopts a social constructivist interpretive framework, assuming that social reality is multiple, processual, and constructed (24). This approach assumes that knowledge is actively constructed through social interactions and shaped by cultural and contextual factors, rather than reflecting a single objective truth.

### 2.2 Study participants

A total of 22 participants from different disciplines were included in this study: 10 EbyO and 12 EbyE.

Initially, a list of 12 leading Dutch participants with knowledge of persistent suicidality was compiled by the authors, containing 10 EbyO and 2 EbyE. However, during the data collection phase, the interviews with EbyE provided unique valuable insights, prompting the inclusion of an additional 10 individuals with lived experience.

The EbyO were purposefully sampled from the authors’ extended networks to hold a broad variety of positions, with some also having held or currently holding dual positions: professor, researcher, psychologist, psychiatrist, medical director, ethicist, spiritual caregiver, philosopher, theologian, and an end-of-life counselor. On average, they had 20 years of work experience.

EbyE were people who self-identified as expert by experience in persistent suicidality. Convenience sampling was used to include participants from this hard-to-reach population. Recruitment took place through the social media channels of various foundations that support people with death wishes and suicidality namely, 113 Suicide Prevention, ‘PsychoseNet,’ and Suicide Prevention Center. Inclusion criteria were good command of Dutch, being older than 18 years, and self-identified as having persistent suicidality. Participants were excluded if they were non-Dutch speaking or if, during the preparticipation telephone interview with psychiatrist SvV, a shared decision was made that participation posed too much of a burden on the participant. In one instance, an individual with lived experience was excluded from participating in the study due to psychological distress.

Participation involved no direct benefits other than contribution to the science.

### 2.3 Data collection

The topic guide for the semi-structured interviews was prepared by the authors, originating from the disciplines of (care) ethics, psychiatry, psychology, and health sciences, and all actively engaged in (research on) suicide prevention and mental healthcare. One of the authors brought lived experience with persistent suicidality in the past. The topic guide included open-ended questions designed to align with the research objectives (Appendix 1). Following the interviews with the initial 12 participants, the topic guide was slightly revised for 10 additional EbyE. The focus was shifted from an observed understanding of persistent suicidality to a greater emphasis on firsthand experiences and needs (Appendix 2).

As data collection took place during the COVID-19 pandemic, the interviews were conducted either online via Microsoft-Teams or in person at a mutually agreed location, based on the participant’s preference. Five of the 22 interviews were conducted in person and the remainder were online. Data collection occurred between May 2021 and April 2022. During the interviews, only the participant and the interviewers (EE or MP, both women and affiliated with 113 Suicide Prevention at the time) were present. EE and MP are trained qualitative researchers and conducted the first two interviews together to ensure a consistent approach. Interviews were conducted in Dutch and fieldnotes were taken. With the participant’s permission, an audio or video recording was made and later manually transcribed verbatim. To minimize their own influence on participants’ answers during the interview, the interviewer posed open-ended and non-directive questions. Additionally, the interviewer adopted the terminology used by the participants to better align with their experiences. Participants were interviewed for the duration of approximately one hour.

### 2.4 Data analysis

Data analysis was conducted by means of reflexive thematic analysis, as described by Braun and Clarke. This approach was first described in 2006 (25) and has been further refined in subsequent publications (26, 27). We followed the steps outlined in the original article and consulted subsequent work to align with the contemporary approach. This method was selected as it aligns with a constructivist framework as the analytic outputs are constructed from codes and through researchers’ active engagement with the data (28). To enhance reliability and capture a broader range of themes, data analysis was conducted by two researchers (EE and MP). As a first step, the researchers familiarized themselves with the data by reading and re-reading the interview transcripts, allowing for a deep engagement with the material. Next, during the initial coding phase, the two researchers independently conducted inductive coding of data relevant to the research questions. Both semantic and latent coding were utilized. The initial codes were compared, and through deepening familiarity with the data, the researchers determined which codes contributed to theme development and which could be discarded, resulting in one final list of codes. Next, the two researchers examined the codes for broader patterns, grouping them in potential themes that effectively and comprehensively captured the underlying meanings in the data. Subsequently, the potential themes were reviewed, with special attention for their different properties and subthemes. Lastly, the themes and subthemes were named, after which they were documented (see **Tables 1**, **2**). For the purpose of this article, the quotations were translated into English.

**Table 1:** Perceived causes of persistent suicidality*.

Themes	Sub-themes
Adverse (childhood) experiences	Abuse as a child (emotional, physical and/or sexual)
	(Temporarily) out-of-home placement or adoption as a child
	Parental neglect or unsafe home situation
	Being the victim of bullying
	Death of loved one
	Being involved in a fatal accident
Psychopathology	(Co-occurrence of) various forms of (long-term) psychopathology
Suicidality as a function	Perceiving the option of suicide as a way to regain control
	Suicidality as a way to express pain and ask for help
	Suicidality as a coping mechanism to reduce emotional suffering
	Suicidality as a means of communication
Demoralization	Feeling that treatment is and will remain ineffective
	Having had adverse experiences with past treatment
	Feeling misunderstood and lacking trust in care
	Disengagement from care
	Non-disclosure
	Having a long history of psychiatric care
	Treatment options are perceived as exhausted

*The prevailing view among experts was that persistent suicidality emerged and persisted due to a complex interplay of multiple factors, rather than stemming from a single cause.

**Table 2:** Needs of individuals with persistent suicidality.

Themes	Sub-themes
(Inter)personal	Acceptance of persistent suicidal thoughts (both for the individual and their loved ones)
	Discussing suicidal thoughts with loved ones
Therapeutical	Discussing suicidal thoughts within therapy
	*- Exploring the meaning of the death wish*
	*- Focusing on living with a death wish, rather than solely emphasizing recovery*
	*- Maintaining autonomy*
	Addressing underlying psychopathology
	Reducing ignorance and stigma among caregivers
	Continuity of care and a safe therapeutic relationship
	Multi-disciplinary care and involvement of family
	*- Spiritual counseling*
	*- Professionals with lived experience*
	*- Body-oriented therapy*
	- *Involvement of family members in therapy*
	Discussing the possibility of medical assistance in dying
Societal	Participation and inclusion
	- *Raising awareness of the invisibility of psychological suffering*
	Financial support

In line with the reflexive thematic analysis approach and given the exploratory nature of this study, data saturation was not pursued as an objective (29). Instead, participants were recruited to capture a broad range of perspectives relevant to the research questions. This diversity provided depth and richness, enabling the research questions to be thoroughly addressed.

ATLAS.ti Web (version 7.6.1) was used for data analysis.

### 2.5 Ethical and safety considerations

The Medical Ethical Review Committee of Amsterdam University Medical Center issued a non-WMO statement for this study (METC number 2021-0225). Prior to the interview, participants received an information letter and written informed consent was obtained. For the EbyE specifically, apart from the preparticipation interview with psychiatrist SvV, the possibility of receiving aftercare was provided. One participant made use of this aftercare. Approximately three days after the interview, narrative interview reports were sent to the participants for comments and/or correction by email. Most found the report complete, with a few suggesting minor corrections or preferred phrasing changes. Additionally, immediately following the interviews, participants explicitly stated that their participation was a positive experience. In particular, different EbyE indicated that they valued being able to openly speak about their death wishes.

Given the relatively small sample size and considerable demographic variation, demographic information was omitted from the verbatims to mitigate the risk of participant identification.

### 2.6 Quality criteria

This project was preregistered on the Open Science Framework on October 7, 2022. Identifier: DOI 10.17605/OSF.IO/DMHRV. This study adhered to the guidelines outlined in the Consolidated Criteria for Reporting Qualitative Research (COREQ) checklist for its reporting (30).

## 3 Results

The characteristics of the EbyO and the EbyE can be found in **Tables 3**, **4**, respectively. They represent a diverse group of varying ages, genders, and backgrounds. All but one EbybE reported psychopathology. Most EbyE recalled the onset of their death wish during their early to mid-teens. While about one-third mentioned periods in which their death wish was absent, it always returned. Moments of relief were often linked to falling in love or effective therapeutic conversations. Yet most, even during periods of well-being, described a persistent background presence of suicidal thoughts, fluctuating in intensity but never entirely absent. EbyE expressed little expectation that their death wish would ever fully disappear. Participants emphasized the heterogeneity among individuals with persistent suicidality and how underlying causes, personal histories, and needs may differ.

**Table 3:** Characteristics of experts by occupation (n=10).

Gender	Participants (n)
Women	4
Men	6
Age (average 54 years)	Participants (n)
25-34	2
35-44	1
45-54	1
55-64	2
65+	4
(Former) occupation *	Participants (n)
End-of-Life Counselor	1
Ethicist	1
Medical-Director	2
Philosopher	2
Psychologist	5
Psychiatrist	2
Professor	1
Researcher	2
Spiritual Counselor	1
Theologist	1
Work experience (average 20 years, range 5–40 years)	Participants (n)
0-9	2
10-19	3
20-29	2
30-39	1
40+	2

*Most experts held more than one occupation.

**Table 4:** Characteristics of the experts by experience (n=12).

Gender	Participants (n)
Women	9
Men	3
Age (average 46 years)	Participants (n)
18-24	1
25-34	2
35-44	1
45-54	5
55-64	2
65+	1
Age of realization of having a death wish *	Participants (n)
Before adulthood	9
During adulthood	3
Current partner status	Participants (n)
Partner	5
Divorced	3
No partner	4
(Step)Children	Participants (n)
(Step)children	5
Childless	7
Working status	Participants (n)
Working	6
Studying	5
Not working or studying	1
Lifetime psychiatric disorders mentioned by participants (average 2.7 disorders, range 0–6 disorders) ** ˟	Participants (n)
Addictive Disorder	1
Autism Spectrum Disorder (ASD)	2
Attachment Disorder	2
Attention Deficit Hyperactivity Disorder (ADHD)	1
Anxiety Disorders	1
Bipolar Disorder	4
Borderline Personality Disorder (BPD)	3
Dissociative Identity Disorder (DID)	1
Dysthymia	1
Eating Disorder	1
Major Depressive Disorder (MDD)	4
Narcissistic Personality Disorder (NPD)	1
(Complex) Post Traumatic Stress Disorder (PTSD)	9
Psychotic Disorder	1
Sleep-Wake Disorder	1

*Some participants expressed always having found life difficult and feeling indifferent from others as a child, but not having had the actual realization of having a death wish until later in life.

**Participants listed several mental problems that are not a psychiatric disorder (i.e., generational trauma inheritance, burn-out, high intelligence, and auditory hallucinations).

**˟**Most participants listed multiple disorders.

In the first part of the results section the insights regarding the perceived causes of persistent suicidality are given. The second part presents themes related to the needs of individuals with persistent suicidality. Quotations from the interviews are included to illustrate how findings are grounded in the data. For each quotation, the number of participant and expert group are given (e.g. EbyE1 for expert by experience, participant 1).

### 3.1 Perceived causes of persistent suicidality

Participants expressed that the persistent suicidality arises from and is sustained by a complex interplay of numerous factors, including biological, psychological, and social influences. These factors differ from person to person and influence an individual’s ability to cope with distress and the weight of the burden they have to bear (i.e. their suffering). EbyO explained that the degree to which people can tolerate suffering varies, and that as long as hope for improvement of their situation exists, people can endure a lot. However, when the experienced burden surpasses one’s capacity to bear it, this can result in a loss of perspective and a sense of despair, leading to thoughts of death. **Table 1** provides an overview of the themes identified regarding the perceived causes of persistent suicidality.

#### 3.1.1 Adverse (childhood) experiences

A frequently mentioned psychosocial factor at the root of persistent suicidality was adverse (childhood) experiences, often resulting in trauma. This was evident in interviews with EbyE, where all but one participant recalled negative events and experiences from their early youth or adolescence as cause of their persistent suicidality. These events included abuse (emotional, physical, and/or sexual), death of a loved one, and (temporary) out-of-home placements. Reported experiences were having a “narcissistic parent,” neglect, being adopted, and having been bullied. EbyO suggested that as a result of these experiences during development, individuals may lack the foundation upon which to build a meaningful and fulfilling life, leaving them without a sense of purpose and consequently developing persistent suicidality. This was evident in the account provided by one of the EbyE regarding the origin of her persistent suicidality:

I believe this is part of why I do not have that joy of living. From childhood, I was told that I am not wanted, that [my mother] would also rather I die by suicide, that I was a burden to others, and that I could not do anything right. That I do not live up to the ideal for her, what she thought it would entail to have a child. [ … ] You grow up with that, and while I can rationally reason that they might be all wrong, I do not know any better than I have been told [these things] every day (EbyE9).

According to the EbyE, childhood experiences have shaped her self-perception, and this has resulted in an inability to relate appropriately to the reality in which she finds herself today. This experience is echoed by the other EbyE who all reported to struggle with low self-esteem or a poor sense of self, leading to a lack of direction, feelings of being a burden to others, and (overly) critical thinking, ultimately contributing to persistent suicidality.

#### 3.1.2 Psychopathology

Psychopathology was often listed as a factor substantially increasing the risk of persistent suicidality. All but one EbyE had a history of psychopathology (see **Table 4**). Notably, nine out of 12 EbyE reported (complex) post-traumatic stress disorder (PTSD). In addition, several EbyE described experiences of self-harm during their interviews. EbyO expressed that many individuals experiencing persistent suicidality face complex long-term psychopathology, often with a history of multiple psychiatric disorders, which imposes a significant burden of suffering. They named mood disorders (depression, bipolar disorder), anxiety disorders, (complex) PTSD, obsessive-compulsive disorder (OCD) or a combination thereof in relation to the occurrence of persistent suicidality. Borderline personality disorder (BPD) and psychotic disorders were identified as conditions where individuals may lose touch with reality, which can lead to recurring suicidal tendencies. Besides, autism spectrum disorder (ASD) was frequently cited in relation to persistent suicidality. EbyO explained that individuals with ASD often tend to think in absolutes, overlooking nuances. As a result, they can experience heightened suffering and engage in impulsive actions. Moreover, people with ASD often feel different or disconnected from others, feeling as if they do not belong. Particularly EbyE with PTSD or attention deficit hyperactivity disorder (ADHD) linked their psychopathology to their persistent suicidality.

#### 3.1.3 The function of suicidality

For multiple EbyE, their suicidality appeared, whether consciously or unconsciously, to have taken on a specific meaning or function in their lives. EbyE reported experiencing significant distress due to negative emotions, self-defeating thoughts, and ongoing external stressors, such as financial debts, relationship breakdowns, or the loss of loved ones. They frequently expressed a sense of needing to escape when confronted with these thoughts or stressors. In this context, both EbyE and EbyO have outlined how persistent suicidality can exist as a “coping mechanism,” viewing it as strategy to evade the hardships of life by altering thoughts and feelings. By perceiving the ability to end their lives as a means to escape and avoid facing inner pain, individuals made their existence more bearable and experience a sense of control. Though counterintuitive, one EbyE even referred to her persistent suicidality as a “survival mechanism” (EbyE10). Another EbyE further clarifies this by stating:

I actually envision this suicidality as sort of similar to addiction. It is a way to escape. To not have to feel. To just want to get away from the pain underneath [ … ]. And it is also a sort of protective mechanism almost, not to have to face the deep pain of abandonment, of not belonging, of not being good enough (EbyE4).

Additionally, several EbyE expressed wanting to communicate their pain and thoughts but not knowing how to do so. This is prevalent in the narrative of one of the EbyE who referred to his first suicide attempt as “a cry for attention, a cry out of desperation” (EbyE11). Some EbyO believed that suicidality can (unconsciously) function as a form of communication, which then becomes a factor contributing to its persistence. This was particularly noted in relation to individuals with BPD. In their view, thoughts of suicide begin because individuals find something deeply painful. Following this, the way their environment reacts to their suicidality can reinforce it, eventually turning it into something that can be used consciously or unconsciously. Therefore, displaying suicidality may become a way for individuals to express themselves, with an underlying plea for help. Although numerous EbyE described expressing their suicidality as a plea for help, they did not particularly link it to the persistence of their suicidality. The functionality of suicidality was understood as a factor bolstering and sustaining it.

#### 3.1.4 Demoralization

Interviews with EbyE revealed that most have long treatment histories, but many reported that these treatments did not yield their preferred results. Feeling that their condition persisted despite their efforts, or following negative experiences with health care professionals regarding suicidality, individuals often reported treatment fatigue. Relatedly, EbyO noted that caregivers may feel powerless when unsure how to proceed with care. Several EbyE shared that their caregivers expressed an inability to find additional ways to treat them, leaving them with the feeling that improvement was no longer attainable. In line with this, participants opposed the use of the term ‘chronic suicidality’, arguing that it conveys a sense of permanence that undermines hope for recovery. Additionally, some EbyO shared that, in their view, treatments are sometimes poorly delivered. This may result in poor treatment outcomes and, consequently, the persistence of suicidality.

Due to demoralization and treatment fatigue, some EbyE had chosen to disengage from the conventional mental health care. Others continued to receive care but chose not to fully disclose their experiences, believing that sharing would not lead to meaningful improvement or due to disagreements with physicians’ past actions and fear of those actions being repeated, as illustrated in the following quotation:

EbyE12: [There] are also a lot of topics that I deliberately do not discuss with [mental health caregivers], because I know that could lead to another admission, or whatever. While it is not necessary, at that moment [ … ]

Interviewer: What are some other topics that you feel you cannot discuss?

EbyE12: [ … ] past traumas or whatever. So, you leave that out, too. Because, oh yes, that can affect me a lot, so if I am affected, they can see that it is going badly, so then, that … Constantly that little thing so of what can I tell, what can I not tell. [ … ] I think the death wish is the number one thing in that, that you just absolutely do not discuss that, because you do not know what the next steps will be.

Interviewer: Do you believe that [the care] offered to you right now is helpful then?

EbyE12: No, I think they intervene too late every time. The ability to recognize the signals seems to be lost these days.

By not being fully open about their experiences, individuals may not receive the care they truly need. This may prevent them from addressing the underlying issues they face, allowing their suicidality and other mental health problems to persist.

### 3.2 Needs of individuals with persistent suicidality

Needs of individuals with persistent suicidality were identified across (inter)personal, therapeutic, and societal levels. **Table 2** provides an overview of the themes identified regarding the needs of individuals with persistent suicidality.

#### 3.2.1 (Inter)personal support

##### 3.2.1.1 Acceptance of persistent suicidal thoughts

A common need highlighted by almost all participants was the acceptance that their suicidality might not disappear over time. Several EbyE reported feeling as if they were not allowed to have these thoughts and were puzzled by their presence, given that they experience thoughts of death alongside feelings of belonging, falling in love, and finding joy in life. One participant explained: “I have a girlfriend, I have my parents, I have my job. I work at [organization]. I have everything … Am I even allowed to be suicidal, I wonder?” (EbyE11). In addition to personally accepting their persistent suicidality, they expressed a need for recognition of it from others.

##### 3.2.1.2 Discussing suicidal thoughts with loved ones

Numerous EbyE indicated that personal attention and the ability to discuss their wish to not be alive play a crucial role in the acceptance of these thoughts. Conversations with family members and friends often helped them through tough times. They explained that talking about the desire to die reduced feelings of loneliness and fostered a greater sense of connection to life. The following quote explains why participants place such value on this:

The fact that you can finally talk about it. That is the most important thing. It is mainly a lot of pain. Then you can share it. That it does not matter for a moment that you do not want to be here, that someone understands that. That you do not have to pretend that you do want to be here (EbyE9).

In some accounts, EbyE expressed a fear of losing relationships or burdening others as a result of being open about their suicidal thoughts, with some reporting that this had indeed happened. As a result, some individuals chose to withhold their thoughts and isolate themselves, believing that this action protects both themselves and others.

#### 3.2.2 Therapeutic support

##### 3.2.2.1 Discussing suicidal thoughts within therapy

Besides the need to openly talk about their persistent suicidality with loved ones, EbyE stressed the importance of discussing persistent suicidality in a therapeutic relationship. They emphasized that discussing their suicidality not only entails acknowledgment of its existence but also in-depth explorations of its origin, characteristics, and one’s personal needs. They seek greater emphasis on learning how to manage suicidal thoughts, with a focus on the meaning and acceptance of these thoughts, rather than solely focusing on recovery. Despite this need, several EbyE reported refraining from disclosing their persistent suicidality to caregivers, fearing immediate crisis interventions, such as hospitalization, which they perceived as overly reactive and as undermining their personal autonomy. EbyO noted that openly discussing the desire to die and the underlying reasons can paradoxically encourage individuals to consider living.

##### 3.2.2.2 Treating psychopathology

Participants also emphasized the importance of addressing any underlying conditions and suffering, if present. EbyO shared that this can be achieved through psychotherapeutic treatments and/or medication. Introducing less harmful coping strategies to manage stress and negative emotions, or focusing on alternative forms of communication, can be beneficial. Several participants emphasized the need to focus on identifying and treating problems in early childhood to help prevent more severe psychopathology and persistent suicidality. Some EbyO shared that, in their view, treatments are sometimes not properly carried out because not all necessary steps are followed. This can result in poor treatment outcomes and, consequently, the persistence of suicidality. Besides, the impact of psychopathology on the suicidal process should be considered in treatment. One EbyE noted that, as suicidality can be seen as a contra-indication for certain types of therapy, persistent suicidality hindered her from receiving the care she sought (EbyE9).

##### 3.2.2.3 Reducing ignorance and stigma among caregivers

Several EbyE reported feeling misunderstood in the care system with regard to their persistent suicidality. One shared: “[I was] always told by institutions that my problems were too complex, and yes, that they could not help me. So, I was basically always filled with medication. But not really heard, seen, or actually helped” (EbyE5). Another participant echoed this experience: “I feel that caregivers often … They know the protocol, they hear something that fits the protocol, so they follow the protocol. And they do not look at ‘Well, what is behind this, why is this happening?’” (EbyE12). Both experiences highlight participants’ feelings that their problems must fit certain frameworks to be addressed by caregivers, and their desire for a more personalized approach. EbyO noted significant ignorance and stigma among caregivers regarding persistent suicidality, suggesting that better training could greatly improve care.

##### 3.2.2.4 Continuity of care and safe therapeutic relationship

Given the long-term nature of their suicidality, participants emphasized the importance of continuity in care. Individuals experiencing persistent suicidality often had an extensive treatment history, and frequent changes in caregivers were perceived to disrupt the development of safe and trusting therapeutic relationships, which they often longed for in care. Other needs related to building a strong and safe therapeutic relationship. EbyE felt that knowing more about caregivers would make discussing intimate experiences easier and create a safer environment. They also suggested addressing transference and countertransference. A lack of attention to these dynamics could threaten the therapeutic relationship and lead to negative treatment outcomes.

##### 3.2.2.5 Multi-disciplinary care and involvement of family

Some EbyE reported having positive experiences with body-oriented therapy, spiritual counseling, or expressed a desire to engage in discussions with professionals who have lived experience. Several EbyE shared how focusing on bodily sensations has helped them to access and process their emotions and memories, which had been difficult to reach through traditional talk therapy alone. Conversations with spiritual counselors and professionals with lived experience were perceived as open and without judgment, offering an existential focus that fostered deeper self-understanding. One of the EbyO highlighted the importance of including family members or friends in therapy, particularly when individuals express feeling like a burden to their loved ones.

##### 3.2.2.6 Discussing the possibility of medical assistance in dying

A couple of EbyE indicated that they might request medical assistance in dying (MAiD) in the future, which is legal under strict conditions for psychiatric suffering in the Netherlands (31). One EbyE expressed that discussing the possibility of MAiD might be helpful to fully unravel and openly discuss persistent suicidal thoughts. She asserted that the knowledge of having the option to request MAiD offered her a sense of safety and peace, even if never choosing to act on it. Several EbyO also shared the believe that it must be accepted that the current treatment options are not effective for everyone. They shared that when a death wish is deliberate and consistent, a shared decision may be made to proceed with MAiD.

#### 3.2.3 Societal support

##### 3.2.3.1 Participation and inclusion

On a societal level, participants expressed the importance of participation and inclusion. EbyE shared that their mental health problems excluded them from activities, like attending college or working, which their peers were engaged in and they too aspired to participate in. They indicated that it can be challenging that their persistent suicidality is often invisible to the outside world, highlighting the need for greater awareness of their suffering. EbyE shared that people often underestimate their problems, which only increases the burden of their suffering.

Do you know how complicated it is to get benefits? [ … ] The [organization that provides benefits to people who are unemployed or unable to work] is always pushing you. You know. There is just no understanding for people who feel this way. And I look perfectly fine on the outside. You know. People also think, ‘Oh, it cannot be that bad.’ And that is really complicated. I am not lying in the gutter, and I am not doing foolish things. I am actually … I am really trying my best (EbyE4).

##### 3.2.3.2 Financial support

Relatedly, several EbyE expressed a need for financial support. They reported being unable to maintain steady employment or continue their studies due to their condition, leading to a need for benefits.

## 4 Discussion

Thematic analysis of the data revealed that persistent suicidality is a complex and multifaceted phenomenon, with underlying causes that can vary between individuals. The findings indicate that adverse (childhood) experiences and psychopathology are perceived as root causes of persistent suicidality. Additionally, suicidality was believed to persist as a result of its function in individuals’ lives, such as acting as a coping mechanism or a form of communication (as noted by EbyO). Other perceived contributing factors include treatment fatigue, non-disclosure of problems, and the experience of reaching the limits of available treatment options. Needs related to persistent suicidality primarily involved individuals’ desire to be genuinely heard and to openly discuss their wish to die. This can help foster acceptance of their desire to cease living.

Although the research base on persistent suicidality is still limited, constraining far-reaching comparisons with prior studies, our findings nonetheless reveal some notable points of overlap with the existing literature. Both our findings and prior studies’ findings indicate that persistent suicidality may be linked to feelings of hopelessness, a loss of future perspective, and early life adversity (12, 18, 32). Additionally, individuals with persistent suicidality were found to report more severe psychopathology as compared to those with no history of suicidality or transient suicidal ideation (8, 14, 15). Notably, though not necessary representative, all but one EbyE reported adverse (childhood) experiences and nine of the 12 EbyE reported a lifetime disorder of (complex) PTSD, outweighing any of the other diagnostic classifications. The shattered assumptions theory, a world-view based model of PTSD, offers a possible explanation for these findings. According to this theory, trauma can damage individuals’ belief in a just and safe world and undermine their sense of self-worth (33). As a result, they may experience heightened vigilance toward potential threats and greater awareness of personal mortality (33, 34). This may contribute to the development of suicidal thoughts, as reported in previous empirical studies (35, 36).

Feelings of hopelessness and the belief that persistent suicidal thoughts would not subside were particularly prevalent among EbyE whose mental health counselor had stated that the treatment options were exhausted. This relates to the finding that the EbyE opposed the phrasing ‘chronic suicidality,’ as it implies the expectation that the wish to no longer be alive will not fade. This is an important finding in the ongoing debate surrounding the definition and classification of suicidality (7, 37). While some scholars suggest distinguishing between acute and *chronic* suicidality, our findings indicate that this phrasing may inadvertently foster feelings of hopelessness. Therefore, we recommend using the term *persistent* suicidality rather than chronic suicidality.

The finding that suicidality may persist because it becomes a form of coping aligns with a previous study of Hennings (38). He described the connection between cognitive reinforcement mechanisms and the persistence of suicidality (38). Drawing parallels between the functions and background of non-suicidal self-injury (NSSI) and building on Hayes’s theory on Experiential Avoidance (39), he provides an explanation of how thought schemes play a significant role in understanding why suicidality may endure in persons with BPD. Similar to non-suicidal self-injury (NSSI), eating disorders, or substance abuse, which can serve as a means to escape certain experiences, suicidality can be viewed as a method to suppress and control emotions. Suicidal thoughts and behaviors can temporarily relieve feelings of emotional pain or offer a sense of control, reinforcing the belief that suicide is a viable solution to distress. As a result, suicidality may develop into a learned method of problem-solving, acting as a reinforcer that strengthens the behavior each time it is resorted to, and thereby encouraging the repetition of this behavior in the future. Unlike classical reinforcement models, which emphasize direct conditioning through active engagement in behavior, suicidality involves indirect conditioning, since the individual has never experienced the cessation of suffering through death (38).

A similar psychological mechanism may help explain the finding that some EbyO believe acute suicidality can become persistent as a means of communication as a result of environmental reactions. Earlier research on suicidality in patients with BPD also highlights its role as a means of communication, suggesting that it is often a way of expressing distress (16, 40). Subsequently, environmental responses like attention and affection might unintentionally act as positive reinforcement, further evoking suicidality. This might make it more likely that an individual will resort to suicidality again in future crises. Accordingly, it has been argued that approaches designed to manage acute suicidality are generally not appropriate for individuals with persistent suicidality (16, 41). In order to manage persistent suicidality, it should be regarded as a phenomenon that may serve a function or provide meaning in the lives of those who experience it. Effective care strategies may include therapies focused on learning alternative coping and communication methods, as well as changing negative thought patterns, for example dialectical behavior therapy.

Across the various identified themes, both those concerning the perceived causes of persistent suicidality and those related to care needs, it becomes apparent that care for individuals with persistent suicidality is at times perceived as ineffective or even harmful. Several EbyE reported feeling misunderstood in the care system, perceiving that caregivers are primarily focused on adhering the protocols rather than truly seeing and understanding them. EbyO similarly indicated that caregivers may lack sufficient awareness and may hold stigmatizing attitudes regarding persistent suicidality. Various caring theorists have warned that (unintentional) deficiencies in caregiving, as experienced by patients, can be harmful for the patients (42). Eriksson identified ‘suffering related to care’ as one of three primary forms of suffering, alongside suffering related to illness and suffering related to life (43). Martinsen distinguishes between two types of the clinician’s gaze, namely the ‘recording’ eye and the ‘perceiving’ eye (44, 45). The recording eye systematizes and differentiates, thereby (unintentionally) lacking openness to the patient’s lived experience. In contrast, the perceiving eye is characterized by openness, allowing the caregiver to be emotionally ‘touched’ by and engaged with the patient. She argues that caregivers (and healthcare systems) are responsible not only to provide technically correct care, but also care that is compassionate and person-centered (44, 45). Following this theoretical framework, care for individuals experiencing persistent suicidality requires precise clinical action guided by the ‘recording eye,’ alongside compassionate engagement through the ‘perceiving eye.’ The systems in which caregivers work should facilitate this.

An important factor to consider in relation to persistent suicidality, though difficult to observe due to its unconscious nature, is transference in therapy. Transference can unconsciously hinder the therapeutic process, contributing to persistence of suicidality. Research underscores the harmful effects of unawareness of transference in suicidal patients (46, 47). Unconscious transference may lead to reenactment of previous trauma (46). Additionally, countertransference, where therapists respond based on their own beliefs, can also negatively impact therapy (46–50). When transference-countertransference dynamics are not properly considered, there is a risk of wrongly and prematurely labeling patients as ‘treatment-resistant’ (46). However, when recognized and understood, both transference and countertransference, were found to be a source of insight into the patient’s experience as they helps reveal unconscious patterns of behavior and emotion (47, 48). Psychodynamic psychotherapy trainings may help healthcare professionals increase their ability to recognize and manage transference and countertransference, leading to improved care for patients with persistent suicidality. A psychotherapist can be involved in treatment when the desired treatment outcomes are not attained.

A key need for individuals with persistent suicidality is the ability to openly discuss their suicidal thoughts, yet some hesitate to do so due to fears of ineffective treatment or (coercive) crisis intervention. This aligns with previous findings that 70% of psychotherapy clients conceal suicidal ideation to avoid unwanted consequences like involuntary hospitalization (51). Relatedly, a study on psychiatrists’ experiences found that, despite acknowledging they cannot prevent all suicides, many felt obligated to keep patients alive, leading to worry and fear that often overshadowed patient-doctor dialogue (52). However, scholars have highlighted that safety-focused responses to suicidality, such as hospitalization, have proven ineffective and may even reinforce suicidal tendencies in patients with persistent suicidality (53–55). A review of 13 studies suggests that open discussions about suicidality can improve mental health outcomes for those seeking treatment (56). These findings highlight the need to encourage patients to disclose suicidal thoughts to receive adequate care. Clinicians may be able to support this by clarifying confidentiality and being transparent about admission procedures, fostering a sense of control and predictability. They should also consider both short- and long-term risks when caring for a persistent suicidal patient, as forced admission may damage trust, worsen suicidality, and hinder ongoing treatment. Additionally, clinician training should focus on fostering open discussions about suicidality and accepting that its diminishment may not always be feasible.

Continuity in care relationships was another need, with previous studies supporting its potential positive impact. A meta-synthesis of 25 qualitative studies on patients’ perceptions of care continuity found that patients viewed relational continuity as contributing to higher quality consultations, characterized by improved mutual understanding and greater comfort (57). A recent systematic review, including 17 studies, found higher relational continuity of care can be beneficial for patients with severe mental illness (58). Moreover, the findings of this review suggest that relational continuity of care may help prevent suicide (58). Although no study has specifically focused on continuity of care for individuals with persistent suicidality, these previous findings appear promising for this group. This finding is also important within the framework of transference–countertransference dynamics, as aversion toward a patient, a manifestation of hatred, may tempt therapists to discontinue a therapeutic alliance (50). Yet it is this very discontinuity of care that places the patient at increased risk of suicide, highlighting the potentially dire consequences of enacting countertransference aversion.

One way to address the need to focus on the meaning and acceptance of persistent suicidality, rather than primarily on recovery, is through a palliative care approach. Researchers have highlighted the significance of palliative care for psychiatric patients (59). While palliative care in psychiatry is still in its early stages, an example of such an initiative is Reakiro in Belgium (60, 61). This facility complements professional care by supporting individuals with a desire for MAiD due to unbearable psychological suffering from a psychiatric disorder, as well as their loved ones. Prior research findings highlight the importance of creating a space to discuss death wishes and the option of MAiD, supporting the idea that by allowing an autonomous choice to die the possibility of choosing life may also emerge (62, 63). Conversely, other study findings raised concerns about MAiD, expressing worries that presenting MAiD as an option might inadvertently discourage patients from pursuing treatments that might benefit them (63–65). Some argued that the role of caregivers should be to support patient in making their lives livable (64). Further research is needed to examine the impact discussing MAiD with persistently suicidal individuals.

### 4.1 Strengths and limitations

This study has several strengths and limitations. As one of the first studies to center on persistent suicidality, this research uniquely contributes to the field by qualitatively examining the phenomenon through interviews both experts with occupational and lived experience. It therefore combines holistic clinical understandings of the phenomenon with firsthand knowledge from lived experience, leading to rich, contextualized data. Besides, this combination of participants ensures that the findings of the research can be generalized to a wider population as multiple viewpoints have been included, reducing information bias. However, this study also has some limitations. Because EbyE participants were recruited through convenience sampling, a substantial proportion originated from the same network, which may have introduced selection bias. Moreover, recruiting EbyE participants through convenience sampling may have introduced a bias toward those more willing or able to articulate their experiences, potentially excluding less vocal perspectives Although the participants were experts on the topic and came from diverse backgrounds, this study is based on the views and experiences of a relatively small group of individuals that were willing to participate. Finally, because the data analysis process involved constant comparison and interpretation, relying heavily on the researchers’ interpretations, some subjective bias is inevitable. Efforts were made to minimize these effects by discussing the findings within the research group, stating positionality, and remaining closely tied to the data. This study was conducted in The Netherlands, where suicide and MAiD (only when strict criteria are met) are legally permitted. As a result, researcher’s interpretations and participants’ experiences may have been shaped by this relatively distinctive context, which should be considered when extrapolating these findings to other countries.

## 5 Conclusion

This study enhances the understanding of persistent suicidality as a complex and multifaceted phenomenon. While the specific perceived causes of persistent suicidality vary among individuals, the findings suggest that it is linked to adverse experiences and psychopathology. It may persist as a coping mechanism, a form of communication, or due to unsuccessful treatment. Key needs include feeling genuinely heard and openly discussing suicidal thoughts. Attention should be given to reasons for non-disclosure and transference-countertransference dynamics in therapy. Findings underscore a need for psychiatric palliative care initiatives and continuity in care relationships. Care should not focus solely on recovery but also emphasize managing and acceptance of suicidal thoughts.

## Data Availability

Data cannot be shared publicly because of to potentially identifying information and lack of participant consent for sharing the anonymized data. The transcripts are archived on a secured server of Amsterdam UMC. Anonymized excerpts of transcripts are available upon request for researchers who meet the criteria for access to confidential data. Requests to access the datasets should be directed to m.m.palm@amsterdamumc.nl.
