# Supplementary material for: Wavering between life and death: a qualitative study into the perceived causes and needs of persons with persistent suicidality
Source: Front Psychiatry. 2025 Oct 24;16:1664180. doi: 10.3389/fpsyt.2025.1664180 (PMC12593954; doi:10.3389/fpsyt.2025.1664180)
Supplement: Supplementary file 1 [file Table1.docx]

Appendix 1. Topic guide for experts by occupation

| **Topics** | **Example questions** |
| --- | --- |
| *Factual data* | Age of expert  Gender of expert  Background of expert (Profession, years of work experience, education level, marital status?) |
| *Exploring suicidality* | What do you understand persistent suicidality to be? |
| From this point forward, the terminology chosen by the individual with lived experience is used, regardless of any psychiatric classifications or context. | |

| *Origin of persistent suicidality* | How do you think persistent suicidality occurs and what can cause it? What factors are related to it? Can you explain this with examples?  How do you think persistent suicidality differs from more other (more acute) forms of suicidality? |
| --- | --- |
| *Course of persistent suicidality* | How does persistent suicidality developed during a life?  - Is it continuously present? Is it always present to the same degree?  What causes it?  Do you think persistent suicidality can ever fully disappear?  How does it manifest itself (thoughts, behaviours, attempts?)? |
| *Needs of individuals with persistent suicidality* | Looking at your experience, what do you think people who are persistently suicidal need regarding their suicidality?  Are there other things you think might be needed? Can you explain which ones? |
| *Treatment and prevention* | How or to what extent do you think persistent suicidality can be prevented?  In your opinion, is treatment for persistent suicidality possible? What does this involve? |
| *Round-up* | Do you have anything to add?  For participants with lived experience: To what extent did you experience the interview as burdensome? If burdensome: safety protocol starts (advise to contact regular care, point out 113 Suicide Prevention and follow-up call by psychiatrist) |
