# Supplementary material for: Wavering between life and death: a qualitative study into the perceived causes and needs of persons with persistent suicidality
Source: Front Psychiatry. 2025 Oct 24;16:1664180. doi: 10.3389/fpsyt.2025.1664180 (PMC12593954; doi:10.3389/fpsyt.2025.1664180)
Supplement: Supplementary file 2 [file Table2.docx]

Appendix 2. Topic guide for experts by experience

| **Topic** | **Example questions** |
| --- | --- |
| *Factual data* | Age of expert  Gender of expert  Background of expert (Profession, education level, marital status?) |
| *Exploring suicidality* | How long have you been struggling with your suicidality? Can you tell more about it?  Do you feel that you are persistently suicidal? Why yes/no? What is it like? |
| From this point forward, the terminology chosen by the individual with lived experience is used, regardless of any psychiatric classifications or context. | |

| *Origin of persistent suicidality* | How do you think your persistent suicidality occurred and what caused it? What factors are related to it? Can you explain this with examples?  How do you think persistent suicidality differs from other (more acute) forms of suicidality? |
| --- | --- |
| *Course of persistent suicidality* | How has your suicidality developed during your life?  - Is it continuously present? Is it always present to the same degree?  What causes it? Do you think your suicidality can ever fully disappear?  How does it manifest itself (thoughts, behaviours, attempts?)? |
| *Needs of individuals with persistent suicidality* | Looking at your experience, what do you think people who are persistently suicidal need regarding their suicidality?  How do you relate to these needs and your feelings? Are you allowed to have them? |
| *Social environment and interaction* | How do you experience the reactions of your environment? Can you always be honest with your environment?  Do you feel that your suicidal thoughts are allowed to be there in society? How do you experience this?  How do you position yourself in society? |
| *Treatment and prevention* | How or to what extent do you think persistent suicidality can be prevented? In your opinion, is treatment for persistent suicidality possible? What does this involve? |
| *Round-up* | How did you experience this interview? Do you have anything to add?  To what extent did you experience it as burdensome? If burdensome: safety protocol starts (advise to contact regular care, point out 113 Suicide Prevention and follow-up call by psychiatrist) |
